# Self-Mixing Interferometer for Acoustic Measurements through Vibrometric Calibration

**DOI:** 10.3390/s24061777

**Published:** 2024-03-09

**Authors:** Simon Chanu-Rigaldies, Pierre Lecomte, Sébastien Ollivier, Thomas Castelain

**Affiliations:** 1 Ecole Centrale de Lyon, CNRS, Universite Claude Bernard Lyon 1, INSA Lyon, LMFA, UMR5509, 69130 Ecully, France; 2 Ecole Centrale de Lyon, CNRS, Universite Claude Bernard Lyon 1, INSA Lyon, LMFA, UMR5509, 69622 Villeurbanne, France; pierre.lecomte@univ-lyon1.fr (P.L.); sebastien.ollivier@univ-lyon1.fr (S.O.); thomas.castelain@ec-lyon.fr (T.C.)

**Keywords:** optical feedback interferometry, self-mixing interferometry, laser feedback interferometry, acousto-optic sensor, optical feedback factor, linewidth enhancement factor, optical vibrometer, acoustic waveguides, sound pressure level

## Abstract

The Self-Mixing Interformeter (SMI) is a self-aligned optical interferometer which has been used for acoustic wave sensing in air through the acousto-optic effect. This paper presents how to use a SMI for the measurement of Sound Pressure Level (SPL) in acoustic waveguides. To achieve this, the SMI is first calibrated in situ as a vibrometer. The optical feedback parameters *C* and α in the strong feedback regime (C≥4.6) are estimated from the SMI vibrometric signals and by the solving of non-linear equations governing the SMI behaviour. The calibration method is validated on synthetic SMI signals simulated from SMI governing equations for *C* ranging from 5 to 20 and α ranging from 4 to 10. Knowing *C* and α, the SMI is then used as an acoustic pressure sensor. The SPLs obtained using the SMI are compared with a reference microphone, and a maximal deviation of 2.2 dB is obtained for plane waves of amplitudes ranging from 20 to 860 Pa and frequencies from 614 to 17,900 Hz. The SPL measurements are carried out for *C* values ranging from 7.1 to 21.5.

## 1. Introduction

Optical interferometers using laser beams as a light source are useful devices in mechanic and acoustic metrology. They exploit optical interferences to measure variations in the optical path, enabling vibrometric [1,2,3] or acousto-optic measurements [4,5,6,7,8,9]. In the first case, the laser targets a surface whose vibrations induce a variation of the optical path associated with the laser beam. In the second case, the measurement of acoustic waves exploits the acousto-optic effect: when a sound wave propagates, it locally varies the refractive index, which leads to variations in the optical path. Most interferometers use optical parts that must be precisely aligned to combine coherent laser beams and produce interferences. The assembly and adjustment of the different parts can be fastidious for acoustic applications. Disturbances of reference beams or installation effects such as sound wave diffraction on optical parts may also occur. In contrast, the Self-Mixing Interferometer (SMI) [3,10,11,12], also known as Optical Feedback Interferometer (OFI) [12,13,14], is a self-aligned interferometer with only two elements in a single component: a laser diode (LD) and a photodiode (PD). The LD targets a retro-reflective surface which back-scatters part of the emitted photons into the LD cavity, where interferences occur from this optical feedback. This phenomenon causes modulations on the LD power and wavelength according to the optical path variations [10,11]. The latter can be estimated by measuring the LD power using the embedded PD, the current of which is converted into voltage to form the SMI signal. Thus, it is possible to use the SMI both as a vibrometer [3,15,16,17] or as an acousto-optical sensor [12,13,14,18,19].

The sensing of sinusoidal acoustic waves in air has been carried out with the SMI [12,18]. However, few studies were focused on its use for Sound Pressure Level (SPL) measurements. A proportional relationship between the amplitude of the SMI signal and the acoustic pressure of a plane wave measured by a microphone has been observed experimentally [13]. The limits of this relationship are not yet clearly defined [14]. In the previous studies, a reference microphone was mandatory to perform the SMI calibration in situ [13,14]. This limits the interest of such a device compared with the use of a microphone alone.

The objective of the present work is to establish an in situ calibration method without the use of a reference microphone, in particular by using the SMI governing equations. For this purpose, two parameters modelling the optical feedback in the LD must be determined: the LD linewidth enhancement factor α [20] and the feedback factor *C* [21]. These parameters depend on the SMI installation (distance between the LD and the retro-reflective surface, retro-reflective surface nature, laser alignment, etc.). Three feedback regimes can be observed depending on the value of *C*, which is linked to the quantity of photons fed back into the LD cavity: weak feedback (C≤1), moderate feedback (1<C<4.6) and strong feedback (C≥4.6) [22]. In a previous paper, we have shown that the SMI sensitivity to acoustic waves increases with the value of *C* [14]. It is therefore advisable to operate in the strong feedback regime in order to obtain the highest sensitivity and to optimize the Signal-to-Noise Ratio (SNR).

Parameters *C* and α can be estimated from SMI vibrometric signals in air at rest [23,24,25,26,27,28,29,30,31,32,33,34,35]. Among the different methods, some are only effective in weak feedback [23,24,25] and others only in moderate feedback [26,27,28]. In the strong feedback regime, SMI vibrometric signals contain discontinuities which leads to a sawtooth-like fringe structure. This particular shape is not observed for acousto-optic measurements in air as the optical path variations are too small [18]. In fact, acoustic waves in air cause the refractive index to change at a rate of $10^{-9}$ Pa^−1^. For instance, if the distance between the LD and the retro-reflective surface is about 20 cm, an acoustic wave at 112 dB_SPL_ induces an optical path variation of 1.2 nanometers [13], which is three orders of magnitude below mechanical vibrations of a few micrometers [3]. For the measurement of *C* and α in strong feedback regime, the methods can be divided into two categories: phase unwrapping methods [29,30,31,33] and direct SMI signal analysis methods [32,34,35].

Phase unwrapping methods require prior calculation of the laser beam phase from the SMI signal [17]. In these methods, *C* and α are estimated by exploiting the laser beam phase. For example, Yu et al. have estimated *C* between 0.5 and 15 with a relative error below 1% [33]. Orakzai et al. have also developed an iterative method for simultaneously estimating *C* and α regardless of the feedback regime [30]. However, phase calculation is a crucial step that can be difficult to automate when the signal is noisy.

In contrast, direct SMI signal analysis methods avoid the need for phase calculation by directly exploiting discontinuities in the SMI signal. As a result, they are faster to implement than phase unwrapping methods. Using a first-order Taylor expansion of the SMI governing equations, Ri et al. [32] have proposed a method to retrieve analytically the values of *C* and α by exploiting the discontinuities in SMI signal. This method can be used to determine *C* values ranging from 1 to 7 and α from 2 to 4.9. An and Liu [34] trained a neural network with SMI signals to estimate the values of *C* and α corresponding to each of them. After training, this network was able to estimate *C* between 0.1 and 10 and α between 2 and 7 from a given SMI signal.

In the present work, we develop a method inspired by Ri et al. [32] adapted here for a wider range of *C* values. As in [32], the method is based on SMI vibrometric measurements and numerical solving of a set of equations derived from the SMI theory. Whereas the Ri et al. method requires prior signal normalization and cannot estimate *C* when it is greater than 7.5, the present method simultaneously normalizes the signal and estimates *C* between 5 and 20 with a maximal relative error of 0.8% and α between 4 and 10 with a maximal relative error of 7.8%. This paper also includes an experimental validation of this method by measuring acoustic plane waves SPL. The calibration of the SMI highly depends on its configuration and alignment. The presented results show the advantage of performing in situ calibration and SPL measurements sequentially.

This paper is organized as follows: elements of theory on the acousto-optic effect and the SMI are recalled in Section 2. Based on synthetic SMI signals, Section 3 describes the calibration method used to normalize the SMI signal and estimate *C* and α. The experimental setup and protocol used to measure plane waves SPL with the SMI are detailed in Section 4. In Section 5, the method is applied for different acoustic waves and optical settings. The paper is concluded in Section 6.

## 2. Theory

In this section, the theoretical background is reminded: Section 2.1 briefly describes the acousto-optic effect and Ciddor’s model. The latter is used to estimate the refractive index of a medium as a function of thermodynamic parameters such as pressure. The governing equations of the SMI are presented in Section 2.2. From those equations, the link between the values of *C*, α and their influences in the SMI signal, in particular on discontinuities, is formalized in Section 2.3.

### 2.1. The Acousto-Optic Effect

An acoustic wave in air can be described as a pressure fluctuation function of space and time. At position r and time *t* one has:

(1) $$p\left(r,t\right)=p_{0}+p^{\prime }\left(r,t\right),$$

where p(r,t) is the medium pressure, $p_{0}$ is the mean pressure and $p^{\prime }\left(r,t\right)$ is the acoustic pressure. Since the optical refractive index of a gas medium n(r,t) depends on its pressure, it is possible to describe the pressure fluctuation as:

(2) $$n\left(r,t\right)=n_{0}+n^{\prime }\left(r,t\right),$$

where $n_{0}$ is the mean optical refractive index of the medium and $n^{\prime }\left(r,t\right)$ is the fluctuation of the optical refractive index caused by the wave propagation. This interaction is known as the acousto-optic effect.

In air, Ciddor’s model describes the refractive index as a function of the pressure, the temperature, the humidity rate, the ${\mathrm{CO}}_{2}$ concentration and the light wavelength [36]. To describe the refractive index fluctuation $n^{\prime }\left(r,t\right)$ as a function of the acoustic pressure $p^{\prime }\left(r,t\right)$, Ciddor’s model can be approximated as:

(3) $$n^{\prime }\left(r,t\right)=\beta \left(\lambda ,p_{0},T_{0},\phi_{h},c_{{\mathrm{CO}}_{2}}\right)p^{\prime }\left(r,t\right),$$

where λ is the light wavelength, $T_{0}$ is the temperature of the medium, $\phi_{h}$ is the relative humidity and $c_{{\mathrm{CO}}_{2}}$ is the ${\mathrm{CO}}_{2}$ concentration. For laboratory conditions of λ=1309 nm, $p_{0}=1$ bar, $T_{0}=20$ °C, $\phi_{h}=50\%$ and $c_{{\mathrm{CO}}_{2}}=440$ ppm, the value of β is $2.6\times 10^{-9}$ Pa^−1^. In Ciddor’s model, the temperature $T_{0}$ is the most significant parameter for β, with a variation of 0.01 Pa^−1^/°C [37].

### 2.2. The Self-Mixing Interferometer

**Figure 1:**
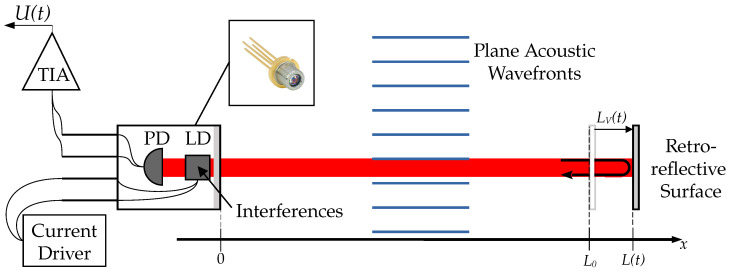
A SMI for acoustic plane waves measurement. LD: Laser Diode. PD: Photo Diode. TIA: TransImpedance Amplifier used to convert PD current into voltage *U*, the SMI signal.

The SMI is based on light feedback dynamics in LDs [10]. When a portion of the emitted photons returns within the laser cavity due to back-scattering from a retro-reflective surface, interferences occurs in the cavity and leads to fluctuations in the LD power and wavelength. A SMI is illustrated in Figure 1, assuming a non-deflected beam.

#### 2.2.1. Optical Path L

From Figure 1, the optical path L is defined as:

(4) $$L\left(t\right)=2\int_{0}^{L(t)}n\left(x,t\right)dx,$$

where *x* is the coordinate along the laser beam, L(t) is its length and n(x,t) is the refractive index. By combining Equations (2)–(4), the optical path L(t) can be rewritten as:

(5) $$L\left(t\right)=2\left(n_{0}L\left(t\right)+\beta \int_{0}^{L(t)}p^{\prime }\left(x,t\right)dx\right)=2n_{0}\left(L_{0}+L_{V}\left(t\right)+L_{\mathrm{AO}}\left(t\right)\right),$$

where $L_{0}$ is the length of the laser beam under static conditions, $L_{V}\left(t\right)$ is the geometric length variations of the laser beam due to displacements of the retro-reflective surface (for instance due to vibrations) such as $L\left(t\right)=L_{0}+L_{V}\left(t\right)$ and $L_{\mathrm{AO}}\left(t\right)=\frac{\beta }{n_{0}}\int_{0}^{L(t)}p^{\prime }\left(x,t\right)dx$ is the apparent change in the laser beam length caused by the acousto-optic effect [13]. Consequently, the SMI is sensitive to the integral of the acoustic pressure along the laser beam. Since the acoustic pressure information is contained in $L_{\mathrm{AO}}\left(t\right)$, only this part of L(t) in Equation (5) needs to be measured to retrieve the acoustic pressure distribution along the laser beam.

#### 2.2.2. Round-Trip Phase Φ

In the case where the photon flux returning back into the cavity is much smaller than that emitted from it, the external round-trip phase shift Φ (simply called phase hereafter) can be described by the following equation [10]:

(6) $$\frac{2\pi }{\lambda_{0}}L\left(t\right)=\Phi \left(t\right)+C\sin\left(\Phi (t)+\arctan(\alpha )\right),$$

where $\lambda_{0}$ is the laser wavelength without optical feedback, L(t) is the optical path outside the laser’s cavity, *C* is the feedback parameter depending on the photon flux back-scattered into the cavity, α is the linewidth enhancement factor [20]. In moderate and strong feedback regimes (C>1), several values for Φ(t) may be solution of Equation (6) for a given L(t), resulting in an hysteresis effect in the behavior of the SMI and discontinuities in Φ [38]. Moreover, Φ(t) at the laser wavelength λ(t) under optical feedback can be written as:

(7) $$\Phi \left(t\right)=\frac{2\pi }{\lambda (t)}L\left(t\right).$$

#### 2.2.3. Feedback Parameter *C*

The feedback parameter *C* is defined as:

(8) $$C=\frac{L(t)}{c_{0}\tau_{\mathrm{in}}}\kappa_{\mathrm{ext}}\sqrt{1+\alpha^{2}},$$

where $c_{0}$ is the speed of light in vacuum, $\tau_{\mathrm{in}}$ is the round-trip time for light in the laser cavity and $\kappa_{\mathrm{ext}}$ is the coupling coefficient depending on the reflectivity of the laser cavity facets and the target [38]. In this study, L variations over time are sufficiently small to treat *C* as a constant. In practice, the $\kappa_{\mathrm{ext}}$ value must not be too large (lower than $10^{-3}$ according to [22]) to avoid an unstable regime known as “coherence-collapse”. According to Tkach et al. [22], this regime could theoretically be reached from a *C* value of about 25 for $L_{0}=0.4$ m.

#### 2.2.4. LD Power P and SMI Signal *U*

The LD power P(t) is modeled as [3]:

(9) $$P\left(t\right)=P_{0}\left[1+m\cos\left(\Phi \left(t\right)\right)\right],$$

where $P_{0}$ is the LD power without optical feedback and *m* is the modulation index such as [39]:

(10) $$m=C\frac{4\tau_{p}c_{0}}{L\left(t\right)\sqrt{1+\alpha^{2}}},$$

where $\tau_{p}$ is the photon lifetime in the laser cavity. In the same way as for value of *C*, variations of L are supposed to be sufficiently small to consider *m* as a constant. In practice, P(t) is measured with an PD embedded in the LD package (see the photograph insert in Figure 1). The PD current, proportional to P(t), is converted into a voltage signal with a TransImpedance Amplifier (TIA) (see Figure 1). Thus, one obtains the SMI signal U(t) from Equation (9) as:

(11) $$U\left(t\right)=U_{0}+\upsilon \cos\left(\Phi \left(t\right)\right),$$

where $U_{0}$ and υ are two constants.

### 2.3. Relationship between C, α and U Signal Shape in Moderate and Strong Feedback Regime

**Figure 2:**
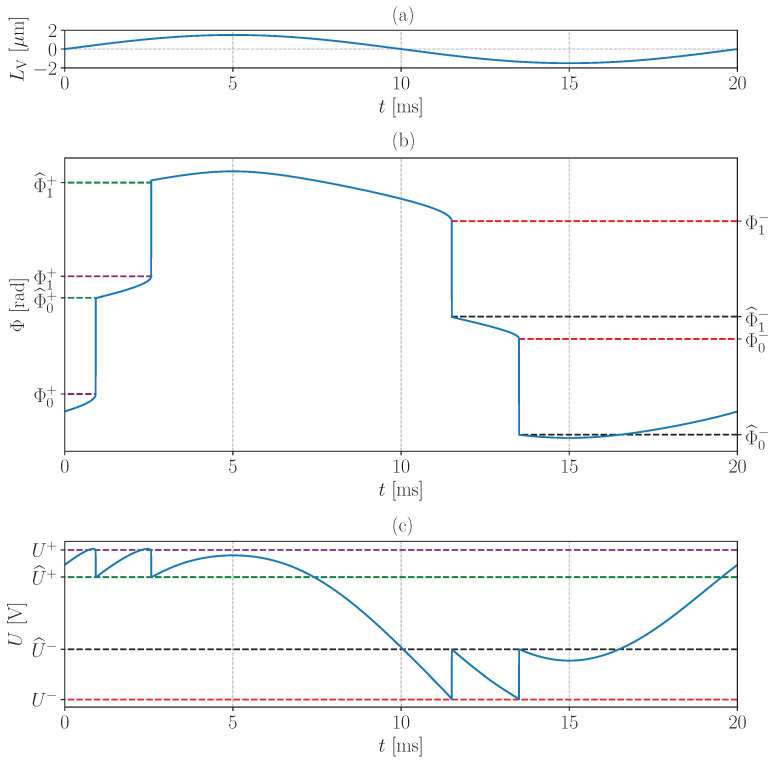
Simulations of Φ and *U* using Kliese et al. algorithm [38] ($\lambda_{0}=1309$ nm, $n_{0}=1.00026$, C=10 and α=6). (**a**) Sinusoidal displacement of the retro-reflective surface $L_{V}$. (**b**) Round-trip phase Φ. The jump values of Φ are indicated by the dashed lines in purple ($\Phi_{k}^{+}$), green (${\hat{\Phi }}_{k}^{+}$), red ($\Phi_{k}^{-}$) and black (${\hat{\Phi }}_{k}^{-}$). (**c**) SMI signal *U*. The jump values of *U* are indicated by the dashed lines in purple ($U^{+}$), green (${\hat{U}}^{+}$), black (${\hat{U}}^{-}$) and red ($U^{-}$).

When performing vibrometric measurements with the SMI in moderate or strong feedback regime, discontinuities can be observed in *U* when variations of L are greater than $\lambda_{0}/\left(2\pi \right)\arccos\left(-1/C\right)$ [38]. Figure 2 shows a simulation of Φ deduced from Equation (6), and of *U* deduced from Equation (11), when $L_{V}$ varies as a sinusoidal function of time ($L_{\mathrm{AO}}=0$ and $L_{0}=0.4$ m in Equation (5)).

$L_{V}$ variations in Equation (5) lead to L and Φ variations in Equation (6). As shown in Figure 2, discontinuities appear in Φ when the latter is increasing and reaches a $\Phi_{k}^{+}$ value, or decreasing and reaches a $\Phi_{k}^{-}$ value, respectively, such as [38]:

(12) $$\Phi_{k}^{\pm }=-\arctan\left(\alpha \right)\pm \arccos\left(\frac{-1}{C}\right)+2\pi \left(k+K\right),$$

with k∈Z. The integer $K=\left\lfloor \frac{2n_{0}L_{0}}{\lambda_{L_{0}}}\right\rfloor$ corresponds to the number of times that the laser phase rotates by 2π along the optical path when $L=L_{0}$. Typically, when $L_{0}=0.4$ m, $\lambda_{0}=1309$ nm, C=10, $n_{0}=1.00026$ and α=6, one obtains K=610,845. The reason for discontinuities in Φ is that the term on the right-hand side of Equation (6) is not monotonic when C>1, which leads to jumps in the values of Φ. For further details, refer to Kliese et al. [38]. Thus, when Φ reaches $\Phi_{k}^{\pm }$, its value jumps to a new value ${\hat{\Phi }}_{k}^{\pm }$ solution of the following equation [38]:

(13) $${\hat{\Phi }}_{k}^{\pm }+C\sin\left({\hat{\Phi }}_{k}^{\pm }+\arctan\left(\alpha \right)\right)=\Phi_{k}^{\pm }+C\sin\left(\Phi_{k}^{\pm }+\arctan\left(\alpha \right)\right).$$

By replacing $\Phi_{k}^{\pm }$ from Equations (12) into Equation (13), one obtains:

(14) $${\hat{\Phi }}_{k}^{\pm }+C\sin\left({\hat{\Phi }}_{k}^{\pm }+\arctan\left(\alpha \right)\right)=-\arctan\left(\alpha \right)\pm \arccos\left(\frac{-1}{C}\right)+C\sin\left(\pm \arccos\left(\frac{-1}{C}\right)\right)+2\pi \left(k+K\right).$$

As Equation (6) may admit several solutions when C≥1, several ${\tilde{\Phi }}_{k}^{\pm }$ values may also be solutions of Equation (14). The ${\hat{\Phi }}_{k}^{\pm }$ value closest to $\Phi_{k}^{\pm }$ is the solution most frequently encountered experimentally [38]. Other solutions relate to the fringe-loss phenomenon that may occur when C>7.8 [40]. This phenomenon is not taken into account in this work because it has not be observed in our experimental setup.

From Equation (11), the discontinuities in Φ are also found in the SMI signal *U*. It is then possible to define $U^{\pm }$ and ${\hat{U}}^{\pm }$ as:

(15) $$\left\{\begin{array}{cc} U^{\pm } & =U_{0}+\upsilon \cos\left(\Phi_{k}^{\pm }\right)\\ {\hat{U}}^{\pm } & =U_{0}+\upsilon \cos\left({\hat{\Phi }}_{k}^{\pm }\right) \end{array}\right..$$

Thus, from Equation (15) the remainder of the Euclidean division of $\Phi_{k}^{\pm }$ and ${\hat{\Phi }}_{k}^{\pm }$ by 2π can be obtained. This remainder corresponds to the $\Phi_{-K}^{\pm }$ and ${\hat{\Phi }}_{-K\mp 1}^{\pm }$ values in Equations (12) and (14). Then, one obtains:

(16) $$\left\{\begin{array}{cc} \Phi_{-K}^{\pm } & =-\arccos\left(\frac{U^{\pm }-U_{0}}{\upsilon }\right)\\ {\hat{\Phi }}_{-K\mp 1}^{\pm } & =-\arccos\left(\frac{{\hat{U}}^{\pm }-U_{0}}{\upsilon }\right) \end{array}\right..$$

By injecting Equation (16) into Equations (12) and (14), respectively, a set of four equations is obtained:

(17) $$\left\{\begin{array}{c} -\arccos\left(\frac{U^{+}-U_{0}}{\upsilon }\right)+\arctan\left(\alpha \right)-\arccos\left(\frac{-1}{C}\right)=0\\ -\arccos\left(\frac{U^{-}-U_{0}}{\upsilon }\right)+\arctan\left(\alpha \right)+\arccos\left(\frac{-1}{C}\right)=0\\ \begin{array}{cc} -\arccos\left(\frac{{\hat{U}}^{+}-U_{0}}{\upsilon }\right) & +2\pi +C\sin\left(-\arccos\left(\frac{{\hat{U}}^{+}-U_{0}}{\upsilon }\right)+\arctan\left(\alpha \right)\right)\\ + & \arctan\left(\alpha \right)-\arccos\left(\frac{-1}{C}\right)-C\sin\left(\arccos\left(\frac{-1}{C}\right)\right)=0 \end{array}\\ \begin{array}{cc} -\arccos\left(\frac{{\hat{U}}^{-}-U_{0}}{\upsilon }\right) & -2\pi +C\sin\left(-\arccos\left(\frac{{\hat{U}}^{-}-U_{0}}{\upsilon }\right)+\arctan\left(\alpha \right)\right)\\ + & \arctan\left(\alpha \right)+\arccos\left(\frac{-1}{C}\right)+C\sin\left(\arccos\left(\frac{-1}{C}\right)\right)=0 \end{array} \end{array}\right..$$

This set of four non-linear equations is used to estimate the four unknowns $U_{0}$, υ, *C* and α from $U^{\pm }$ and ${\hat{U}}^{\pm }$ values, which are measurable in the SMI signal *U*. It is solved using a numerical approach.

Compared with the work in [32], the current approach has several advantages. In fact, in [32] a first-order Taylor expansion of the sine function in Equation (6) is made. After some developments, closed form solutions for *C* (C<7.5) and α from the normalized SMI signal are derived. However, the values of $U_{0}$ and υ have to be estimated beforehand (i.e., the normalization of the SMI signal), which is not the case with the current approach. In addition, the direct resolution of the Equations (17) extends the validity range for *C* as discussed in the following Section 3.

Now that this system of Equation (17) is implemented, the aim of the next section is to apply it to calibrate the SMI.

## 3. Simulation of the Calibration Method

In order to retrieve vibrometric or acousto-optic information with the SMI, it is necessary to estimate the optical path L from the SMI signal *U*. Thus, according to Section 2, the following steps should be carried out:

1. Calibration:
  (a) Measurement of a vibrometric signal *U* with discontinuities,
  (b) Estimation of $U^{\pm }$ and ${\hat{U}}^{\pm }$ from the SMI signal *U*,
  (c) Solving the set of Equations (17) to estimate $U_{0}$, υ, *C* and α.
2. Measurement:
  (a) Measurement of an acousto-optic or vibrometric signal *U* without modifying the SMI configuration,
  (b) Estimation of Φ from *U* with $U_{0}$ and υ in Equation (11),
  (c) Estimation of L from Φ with *C* and α in Equation (6).

**Figure 3:**
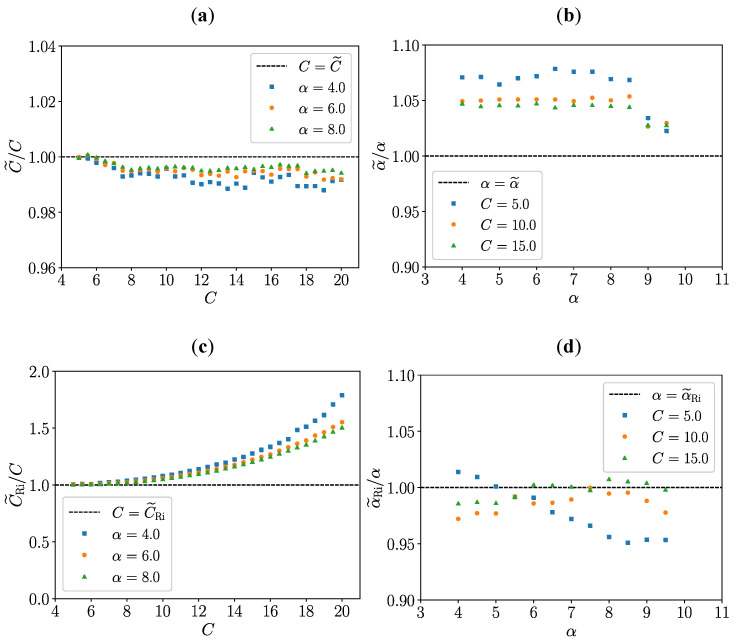
*C* and α estimations. (**a**,**b**) $\tilde{C}/C$ and $\tilde{\alpha }/\alpha$ by solving Equation (17), (**c**,**d**) ${\tilde{C}}_{\mathrm{Ri}}/C$ and ${\tilde{\alpha }}_{\mathrm{Ri}}/\alpha$ with Ri et al. approach [32].

This section presents simulations of the calibration steps 1. (a)–(c). For 1. (a), noiseless SMI signals, similar to those shown in Figure 2, are simulated using the Kliese et al. [38] algorithm with *C* values ranging from 5 to 20 and α values ranging from 4 to 10. The simulations are carried out with $L_{V}\left(t\right)=3.15\times 10^{-6}\cos\left(2\pi 50t\right)$ meters and a sampling rate of 1 MHz. In 1. (b), for each simulation of *U*, the values of $U^{\pm }$ and ${\hat{U}}^{\pm }$ are estimated by computing the temporal derivative of *U* and by using a peak detection algorithm. For 1. (c), the set of Equation (17) is solved using the least-squares method. In the reported cases, the *fsolve* Python function from the *scipy.optimize* library is used with a tolerance of $10^{-12}$. This function uses a numerical method inspired by the Gauss-Newton algorithm to solve non-linear equation systems [41]. Solving the set of Equation (17) yields estimated values for $U_{0}$, υ, *C* and α, which hereafter are denoted $\tilde{U_{0}}$, $\tilde{\upsilon }$, $\tilde{C}$ and $\tilde{\alpha }$, respectively. The values of $\tilde{C}/C$ and $\tilde{\alpha }/\alpha$ are shown in Figure 3a,b as a function of *C* and α, respectively. For comparison, the *C* and α values are also estimated by the Ri et al. method [32]. They are denoted ${\tilde{C}}_{\mathrm{Ri}}$ and ${\tilde{\alpha }}_{\mathrm{Ri}}$, respectively, and ${\tilde{C}}_{\mathrm{Ri}}/C$ and ${\tilde{\alpha }}_{\mathrm{Ri}}/\alpha$ values are shown in Figure 3c,d.

In the following, the maximal relative error $\delta_{\tilde{X}}$ is defined such as:

(18) $$\delta_{\tilde{X}}=\max\left(\frac{\left|\tilde{X}-X\right|}{X}\right),$$

where *X* represents *C* or α.

The estimation $\tilde{C}$ by solving the set of Equation (17) gives results with a maximal relative error of $\delta_{\tilde{C}}=0.8$%. It seems to be barely sensitive to the α value with a better estimation when α increases. This trend is the same for Ri et al. approach when C<7.5. However, as *C* increases beyond, ${\tilde{C}}_{\mathrm{Ri}}$ values deviates from *C* values due to the use of a first-order Taylor expansion [32].

The values of $\tilde{\alpha }$ by solving the set of Equation (17) are slightly overestimated, the relative error does not exceed $\delta_{\tilde{\alpha }}=7.9$% and seems to decrease with the value of *C*. In comparison, the maximum error with the Ri et al. method is 4.9%. In general, the estimates of *C* and α values depend on the correct estimation of the ${\tilde{U}}^{\pm }$ and $U^{\pm }$ values which quantify the discontinuities in *U* signal. Their estimation improves as the sampling rate increases. In the case of noisy signals *U*, an averaging process should be used, as discussed in Section 4.2. Note that the estimation of *C* and α presented here does not require exact knowledge of $L_{V}$, as long as the latter oscillates with an amplitude high enough to observe discontinuities in *U*:$U^{\pm }$ and ${\tilde{U}}^{\pm }$.

Finally, once the values of $\tilde{C}$ and $\tilde{\alpha }$ are retrieved, the SMI measurement of step 2. (a) can be processed by using steps 2. (b) and 2. (c) and the optical path L can be estimated. Moreover, if the variations in L are only caused by acoustic waves, it is possible to estimate $L_{\mathrm{AO}}$ as well as the acoustic pressure in Equation (5) when its distribution along the laser beam is known.

## 4. Experiments

**Figure 4:**
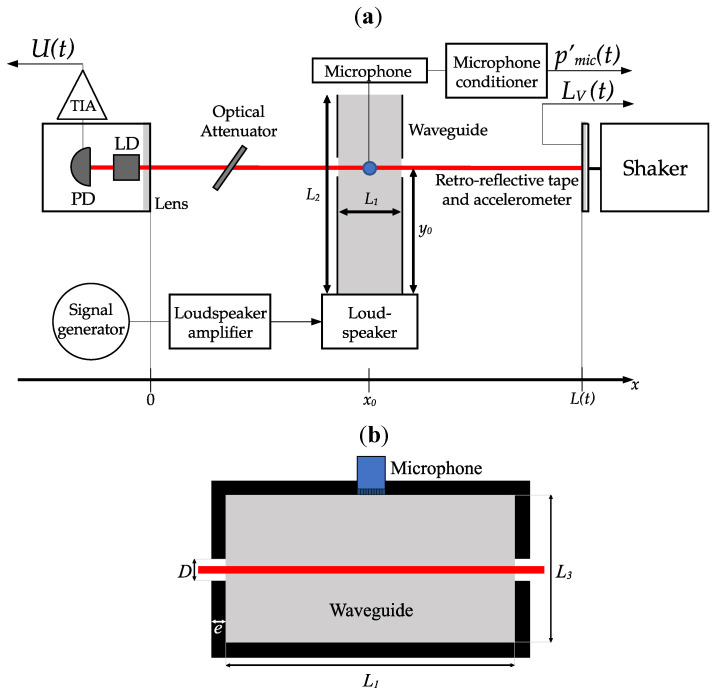
Scheme of the experimental setup. (**a**) Top view. (**b**) Waveguide cross-section through which the laser beam passes.

In this section, an experimental setup (Section 4.1) and a protocol (Section 4.2) are presented to measure the SPL of acoustic plane waves in a waveguide using nothing but a single SMI. The experimental setup is shown in Figure 4. It allows for both SMI calibration and acousto-optic measurements in a sequential manner.

### 4.1. Experimental Setup

As shown in Figure 4, the LD targets a retro-reflective tape mounted on a shaker. When the shaker is turned on and the acoustic source is turned off, it allows for SMI calibration. When the acoustic source is turned on and the shaker is turned off, it allows for acousto-optic measurements. Technical details on this setup are given hereafter.

#### 4.1.1. The SMI

The SMI uses a Thorlabs© L1310P5DFB laser diode (LD) (Newton, NJ, USA) with a wavelength $\lambda_{0}=1309$ nm and a maximum output power of 5 mW. The laser beam is collimated with a Thorlabs© C110TMD-C lens. An embedded PD generates a current proportional to the LD power P. The latter is converted into a voltage *U* through the use of a Femto© DLPCA-200 TIA configured with a gain of $10^{4}$ V/A. The LD is driven by a Thorlabs© LDC205C current driver, and its temperature is maintained at 12 °C by a Thorlabs© TED200C temperature controller.

The laser targets a retro-reflective tape glued on a PCBpiezotronics© 352C65 accelerometer (Depew, NY, USA) placed at $L_{0}=0.4$ m from the laser diode. The accelerometer associated to a PCBpiezotronics© 482C05 conditioner allows for the measurement of $L_{V}$, defined in Equation (5). The accelerometer is mounted on a Brüel & Kjaer© 4810 shaker (Naerum, Denmark) to control $L_{V}$ in order to evaluate the SMI parameters by the method presented in Section 3.

Finally, an optical attenuator Thorlabs© NDL-25C-4 is used to change the amount of photons returning to the laser cavity, which allows for the variation of the feedback parameter *C*.

#### 4.1.2. The Acoustic Source

To generate acoustic waves of different amplitudes and frequencies, two different rectangular waveguides are used below their cut-off frequency. Inner length and wall thickness of the two waveguides together with technical details of the apparatus are presented in Table 1. Each waveguide is excited at one end by a loudspeaker powered by a Visaton© AMP 2.2 LN amplifier. The laser beam passes through the waveguide by two side holes of diameter *D*. For comparison, a microphone without protection grid, connected to a Brüel & Kjaer© NEXUS 2690-A-0F2 conditioner, is flush-mounted on the waveguide wall, in the same section as the one crossed by the laser. It allows for a measurement of the acoustic pressure denoted $p_{\mathrm{mic}}^{\prime }$ after a calibration using a B&K© 4213 calibrator.

For acoustic waves with frequencies below the cut-off frequency, it is assumed that plane waves propagates in the waveguide [42]. Thus, in the configuration of Figure 4 where the plane wavefronts are parallel to the laser beam and neglecting the acoustic radiation through the waveguide side holes, $L_{\mathrm{AO}}$ (see Equation (5)) can be related to the acoustic pressure inside the waveguide $p^{\prime }$ such as:

(19) $$L_{\mathrm{AO}}=\frac{\beta }{n_{0}}\int_{0}^{L}\Pi \left(\frac{x-x_{0}}{2L_{1}}\right)p^{\prime }dx=\frac{\beta L_{1}}{n_{0}}p^{\prime },$$

where Π is the rectangular function and $x_{0}$ the waveguide center along *x* axis. Appendix A presents and discusses simulated results when radiations through the waveguide holes are taken into account.

### 4.2. Protocol

**Figure 5:**
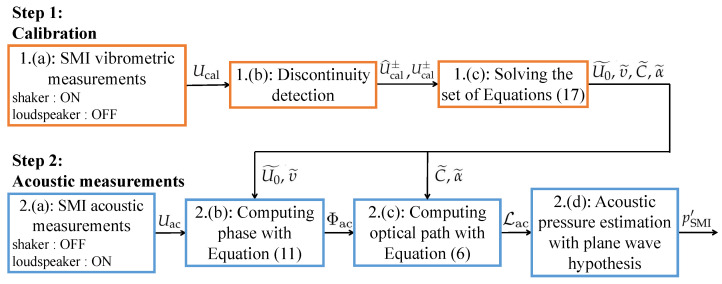
Protocol for acoustic waves measurements with the SMI. The calibration steps are illustrated in orange and the acoustic measurement steps in blue.

The experiments are carried out according to the calibration and measurement steps presented in Section 3 and summarized in Figure 5.

**Figure 6:**
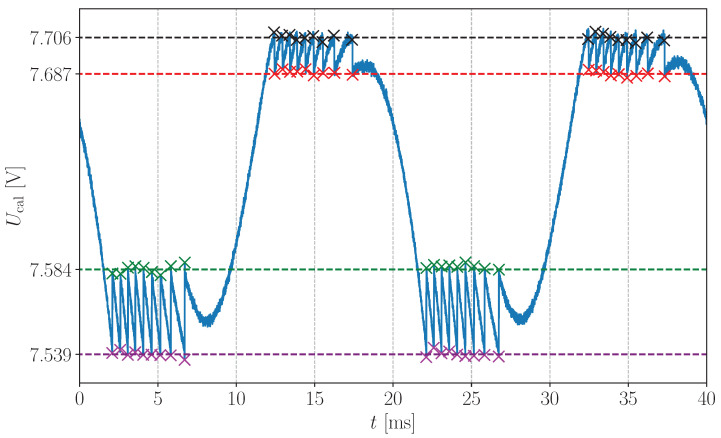
Extract of the SMI signal $U_{\mathrm{cal}}$ used to calibrate the SMI. Values of ${\hat{U}}_{\mathrm{cal}}^{+}$, ${\hat{U}}_{\mathrm{cal}}^{-}$, $U_{\mathrm{cal}}^{+}$ and $U_{\mathrm{cal}}^{-}$ are represented by the dashed lines in red, green, black and purple, respectively. These coloured lines are estimated by averaging the ordinates of the corresponding coloured points.

1. Calibration:
  (a) The SMI is targeting a retro-reflective tape mounted on a shaker through the acoustic waveguide. During this step, no acoustic wave propagates in the waveguide and $L_{\mathrm{AO}}=0$ in Equation (5). The displacement $L_{V}$ is generated by the shaker driven sinusoidally at 50 Hz, as in Section 3. Its amplitude is set large enough to produce a SMI signal, denoted $U_{\mathrm{cal}}$, with around ten discontinuities per period. $U_{\mathrm{cal}}$ is acquired for 2 seconds at a sampling rate of 200 kHz in order to measure 100 periods. An example of measured $U_{\mathrm{cal}}$ signal is shown in Figure 6.
  (b) ${\hat{U}}_{\mathrm{cal}}^{\pm }$ and $U_{\mathrm{cal}}^{\pm }$ are estimated by averaging the ordinate of the points directly before and after each discontinuity as illustrated in Figure 6. This allows to reduce the impact of noise. As in Section 3, the points used for the averaging are estimated by computing the derivative of $U_{\mathrm{cal}}$ and by using a peak detection algorithm.
  (c) $\tilde{U_{0}}$, $\tilde{\upsilon }$, $\tilde{C}$ and $\tilde{\alpha }$ are estimated by solving the set of Equation (17) with the Python function *fsolve* from the *scipy.optimize* library with a tolerance of $10^{-12}$.
2. Acoustic measurements:
  (a) After calibration, the SMI alignment is not modified to avoid any change in the values of *C*, α and υ. Then, the shaker is turned off and the loudspeaker is driven with a sinusoidal signal tuned to one of the waveguide resonant frequencies in order to obtain a high SNR. The resulting SMI signal is denoted $U_{\mathrm{ac}}$. For each acquisition, by varying the sampling rate, one thousand consecutive samples of $U_{\mathrm{ac}}$, $L_{V}$ and $p_{\mathrm{mic}}^{\prime }$ are acquired to capture 100 periods of the acoustic wave with 10 samples per period.
  (b) $\Phi_{\mathrm{ac}}$ is estimated from $U_{\mathrm{ac}}$ with $\tilde{U_{0}}$, $\tilde{\upsilon }$ and Equation (11).
  (c) $L_{\mathrm{ac}}$ is estimated from $\Phi_{\mathrm{ac}}$ with $\tilde{C}$, $\tilde{\alpha }$ and Equation (6).
  (d) Then, $L_{V}+L_{\mathrm{AO}}$ is computed with Equation (5) by taking into account only the alternative component (AC) of $L_{\mathrm{ac}}$. Despite that the shaker is turned off, mechanical vibrations $L_{V}$ may occur during acoustic measurements. To compensate for length variations in $L_{\mathrm{ac}}$, $L_{V}$ is estimated from the accelerometer signals after two temporal integrations and subtracted from $L_{\mathrm{ac}}$. Finally, the acoustic pressure in the waveguide denoted $p_{\mathrm{SMI}}^{\prime }$, is computed using $L_{\mathrm{AO}}$ and Equation (19).

This protocol is applied in the following section.

## 5. Results and Discussions

This section discusses results from two experiments. First, in Section 5.1, SPL measurements with the SMI for acoustic waves of different amplitudes and frequencies are compared to microphonic measurements. Then in Section 5.2, the protocol is repeated for different values of *C* and three SPL at a single acoustic frequency.

### 5.1. SMI and Microphonic Measurements Comparison

**Figure 7:**
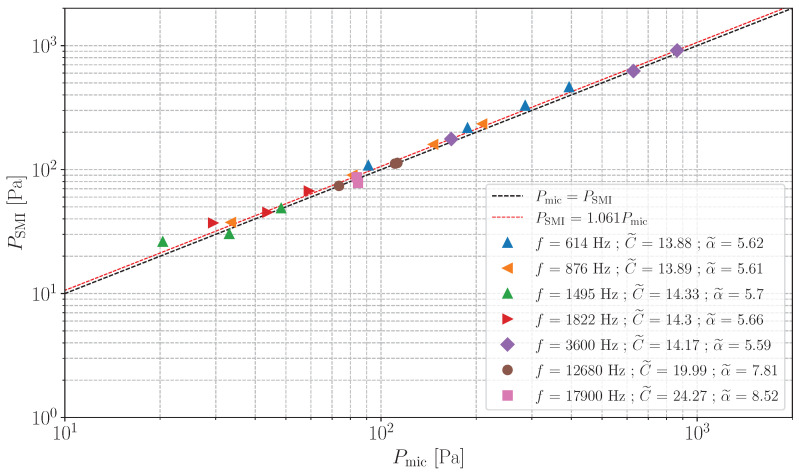
Estimation of SPL obtained by the SMI against the microphone on $P_{\mathrm{mic}}$. The black dotted line shows the straight line with equation x=y. The red dashed line is the linear regression of all the experimental points with $r^{2}=0.97$. The triangle-shaped points are obtained with waveguide No. 1 and the others with waveguide No. 2.

In this section, SPL estimations of sinusoidal acoustic plane waves with the SMI and the microphone are compared. These estimations, denoted $P_{\mathrm{SMI}}$ and $P_{\mathrm{mic}}$ are defined as:

(20) $$P_{i}=\frac{2}{N}\left|F\left(p_{i}^{\prime }\left(t\right)\right)\left[f\right]\right|,$$

respectively, where *f* is the acoustic wave frequency, N=1000 is the number of acquired samples and i∈{SMI,mic}. F is the discrete Fourier transform computed between −5f and 5f with a frequency resolution of f/100. Figure 7 shows the comparison between $P_{\mathrm{SMI}}$ and $P_{\mathrm{mic}}$. Note that for each frequency measurement, the SMI is calibrated following the steps described in Section 4.2. As seen in Section 3, calibration results do not depend on the frequency of the acoustic waves. The results presented here are a compilation of several measurements taken on different days. It was therefore decided to carry out the calibration protocol before measuring each frequency.

The slope of the linear regression between $P_{\mathrm{SMI}}$ and $P_{\mathrm{mic}}$ is 1.061 (0.51 dB) and the maximal difference between $P_{\mathrm{mic}}$ and $P_{\mathrm{SMI}}$ is 2.2 dB. This good agreement confirms the suitability of the SMI for acoustic measurements in waveguides (from 20 to 860 Pa and between 614 and 17,900 Hz in the reported case), using the calibration method presented in Section 3 and the simple model for $L_{\mathrm{AO}}$ in Equation (19).

It may also be noted that the $\tilde{C}$ values estimated in Figure 7 are greater than 7.8. For these values, the SMI may be subject to fringe-loss [40]. This phenomenon is not observed in our experimental configuration. It is important to note that if it were observed, the system of Equation (17) and the calibration method presented in Section 3 would no longer be valid.

### 5.2. Repeating the Protocol for Different Values of C

The aim of the second experiment is to verify that the SMI SPL estimation does not depend on the value of *C* if the calibration method detailed in Section 3 is applied. For this purpose, the value of *C* is modified by the use of an optical attenuator (see Figure 4). Measurements of sinusoidal waves with three different SPL (10, 53 and 400 Pa) are performed. This frequency is fixed at 614 Hz which is a resonance frequency of the waveguide No. 1. The SMI calibration protocol is repeated for each measurement. In Table 2, results are presented and compared to the microphone pressure estimation.

As shown in Table 2, the between difference $P_{\mathrm{mic}}$ and $P_{\mathrm{SMI}}$ never exceeds 2.1 dB and is not correlated with $\tilde{C}$. In the same way that in Section 3 (see Figure 3a), this shows that the calibration method seems to be effective for different $\tilde{C}$ values between 7.1 and 21.5.

## 6. Conclusions and Future Works

An efficient calibration method for measuring acoustic plane waves with an SMI has been detailed. It consists of measuring four parameters of the SMI $U_{0}$, υ, *C* and α in the strong feedback regime (C>4.6) with vibrometric measurements and solving a set of non-linear equations derived from the SMI theory. This calibration method, which does not require comparison with a reference microphone, allows the SMI to be used as an acoustic sensor. SMI measurements of acoustic plane waves in a waveguide have been carried out in the dynamic range from 20 to 860 Pa and at frequencies from 614 to 17,900 Hz. The results of these measurements are similar to those of microphones.

Note that the calibration method presented in this paper uses a shaker (see Section 4) to which the retro-reflective surface is glued. However, the calibration only requires vibrations of sufficiently large amplitude to produce discontinuities in the SMI signal. Vibrations could also be generated by the free oscillations of a 1-degree-of-freedom mechanical system. This system could, for example, be excited by a simple fingertip impact, making the SMI easily deployable for in situ acoustic measurements.

Future work will investigate the use of a SMI as a non-intrusive acoustic sensor and its ability to measure ultrasonic acoustic waves at higher acoustic levels, such as shock waves.

## Figures and Tables

**Table 1:** Characteristics of the waveguides. See Figure 4 for the correspondences of $y_{0}$, *D*, *e* and the waveguide dimensions $L_{1}$, $L_{2}$ and $L_{3}$.

Wave-Guide No.	$L_{1}\times L_{2}\times L_{3}$ (mm)	Side Holes Diameter *D* (mm)	$y_{0}$ (mm)	Wall Thickness *e* (mm)	Cut-Off Frequency (kHz) [42]	Loud-Speaker Model	Microphone Model
1	45×430×25	6	430	10	3.5	Audax© AM130RL0 (Paris, France)	1/4” B&K© 4939
2	10×200×10	3	170	1	18	Eminence© APT80 (Eminence, KT, USA)	1/8” GRAS© 40DP (Holte, Denmark)

**Table 2:** Values of $\tilde{C}$, $\tilde{\alpha }$, $P_{\mathrm{SMI}}$ and $20\log\left(P_{\mathrm{SMI}}/P_{\mathrm{mic}}\right)$ for 614 Hz sinusoidal waves of different amplitudes $P_{\mathrm{mic}}$.

$P_{\mathrm{mic}}$ [Pa]	$\tilde{C}$	$\tilde{\alpha }$	$P_{\mathrm{SMI}}$ [Pa]	$20\log\left(P_{\mathrm{SMI}}/P_{\mathrm{mic}}\right)$ [dB]
10	7.1	6.5	11.3	1.1
	9.0	6.3	9.4	−0.5
	11.5	6.2	12.8	2.1
	16.6	6.3	9.7	−0.3
	21.3	6.6	10.3	0.3
53	7.0	6.0	60.6	1.2
	7.7	5.8	60.7	1.2
	9.9	6.3	58.0	0.8
	15.3	6.5	54.2	0.2
	21.5	6.8	59.2	1.0
400	7.4	6.2	468	1.4
	8.0	6.1	449	1.0
	10.5	6.1	429	0.6
	15.3	6.2	451	1.0
	19.2	6.7	453	1.1

**Figure A1:**
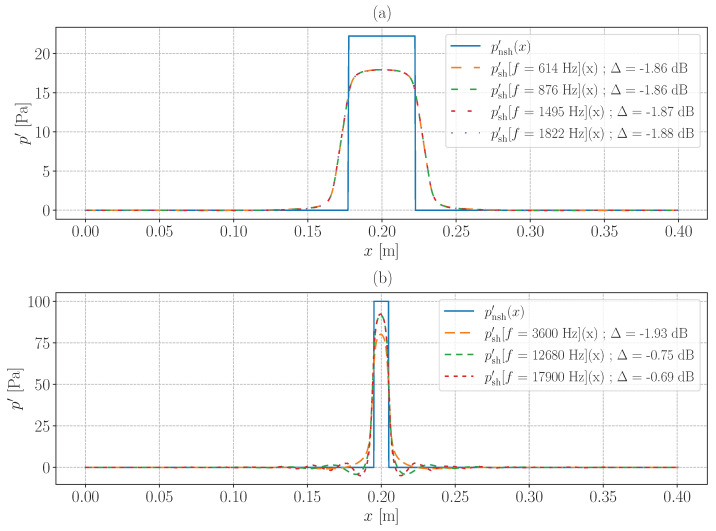
Acoustic pressure $p^{\prime }$ distribution along the laser beam axis *x* at $t_{\max}$. $L_{\mathrm{AO}}=2.6\times 10^{-9}$ m, $n_{0}=1.00026$, $\beta =2.6\times 10^{-9}$ Pa^−1^ and $L_{0}=0.4$ m. (**a**) Waveguide No. 1. (**b**) Waveguide No. 2. Refer to Table 1 for waveguides parameters.

## Data Availability

The data that support the findings of this study are available from the corresponding author upon reasonable request.

## Abbreviations

The following abbreviations are used in this manuscript:

SMI	Self-Mixing Interferometer
LD	Laser Diode
PD	Photodide
TIA	TransImpedance Amplifier
SNR	Signal-to-noise Ration
SPL	Sound Pressure Level

## Appendix A. Acoustic Pressure along the Laser Beam with Waveguide Side Holes Radiation

The SPL estimation in the waveguide using $L_{\mathrm{AO}}$ in Equation (5) requires the knowledge of the acoustic pressure $p^{\prime }$ along the laser beam. In Equation (19), $p^{\prime }$ is considered to be null outside the waveguide and constant inside it. However, in the experiment described in Section 4, the waveguide presents side holes allowing the laser to pass through. Acoustic radiation through these holes modifies $p^{\prime }$ distribution along the laser beam. This Appendix presents simulations of $p^{\prime }$ along the laser beam with and without taking into account the side holes radiations.

Figure A1 displays the acoustic pressure $p^{\prime }$ along the laser beam axis *x* (see Figure 4) with $L_{0}=0.4$ m, $n_{0}=1.00026$, $\beta =2.6\times 10^{-9}$ Pa^−1^ and $L_{\mathrm{AO}}=2.6$ nm in two cases:

- In the case of a plane wave hypothesis (see Equation (19)) with no side holes radiation. This pressure is denoted $p_{\mathrm{nsh}}^{\prime }$ (where subscript “nsh” stands for “no side holes radiation”).
- In the case where side holes radiations are taken into account. The acoustic pressure $p^{\prime }$ is numerically simulated for each frequency *f* of Figure 7, using COMSOL©, by solving the Helmholtz equation with a finite element method [43]. The simulations are carried out with the geometric parameters of Table 1 and the acoustic source is modelled by a 1 m/s velocity source placed at one extremity of the waveguide. From the resulting acoustic pressure, one obtained $p_{\mathrm{sh}}^{\prime }\left[f\right]$ (where subscript “sh” stands for “side holes radiation”) such as:
  (A1) $$p_{\mathrm{sh}}^{\prime }\left[f\right]\left(x,t\right)=\frac{n_{0}p^{\prime }\left(x,t\right)}{\beta \int_{0}^{L_{0}}p^{\prime }\left(x,t\right)dx}L_{\mathrm{AO}}\left(t\right)$$

For both cases, the acoustic pressure is computed at a time $t_{\max}$ when its value at the center of the waveguide ($x_{0}=0.2$ m, see Figure 4) reaches its maximum amplitude.

One denotes Δ the difference in acoustic pressure at the waveguides center at $x_{0}$. Those values are displayed in Figure A1 and are defined by:

(A2) $$\Delta =20\log_{10}\left(\left|\frac{p_{\mathrm{sh}}^{\prime }\left[f\right]\left(x_{0}\right)}{p_{\mathrm{nsh}}^{\prime }\left(x_{0}\right)}\right|\right).$$

As shown Figure A1, $p_{\mathrm{sh}}^{\prime }\left[f\right]\left(x_{0}\right)<p_{\mathrm{nsh}}^{\prime }\left(x_{0}\right)$. In fact, for $p_{\mathrm{sh}}^{\prime }\left[f\right]\left(x\right)$, part of the the acoustic energy is radiated outside the waveguide and one has $\int_{0}^{L_{0}}p_{\mathrm{sh}}^{\prime }\left[f\right]\left(x\right)dx=\int_{0}^{L_{0}}p_{\mathrm{nsh}}^{\prime }\left(x\right)$$dx=L_{\mathrm{AO}}$. The study and modelling of the relationship between radiated pressure and side hole dimensions with respect to acoustic frequency is left to future work [44]. In all the simulated cases, the difference between $p_{\mathrm{sh}}^{\prime }\left[f\right]\left(x_{0}\right)$ and $p_{\mathrm{nsh}}^{\prime }\left(x_{0}\right)$ is below 1.93 dB. We conclude for the measurements carried out in Section 5 that the simple analytical model of Equation (19) is therefore sufficient to estimate $P_{\mathrm{SMI}}$.
